# Supplementary material for: Muscle Strength Training and Monitoring Device Based on Triboelectric Nanogenerator for Knee Joint Surgery
Source: Micromachines (Basel). 2025 Dec 6;16(12):1387. doi: 10.3390/mi16121387 (PMC12735041; doi:10.3390/mi16121387)
Supplement: Supplementary file 1 [file micromachines-16-01387-s001.zip › micromachines-3979685-supplementary.pdf]

Muscle strength training and monitoring device based on triboelectric nanogenerator for knee joint surgery

Jing Liu[1]*, Yi Zhang[2], Xia Liu[1], Chenming Sun[1], Youquan Wang[1]

[1] Faculty of rehabilitation medicine, Jining Medical University, Jining, 272067, China.

[2] Faculty of Electrical and Electronic Engineering, Changchun University of Technology, Changchun, 130012, China.

Support pictures

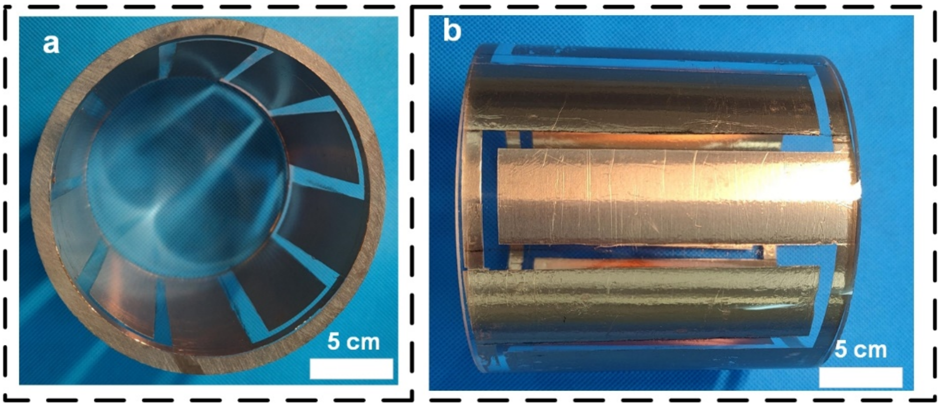

Figure S1. (a) MSTKJS-TENG copper electrode top view, and (b) MSTKJS-TENG copper electrode front view.

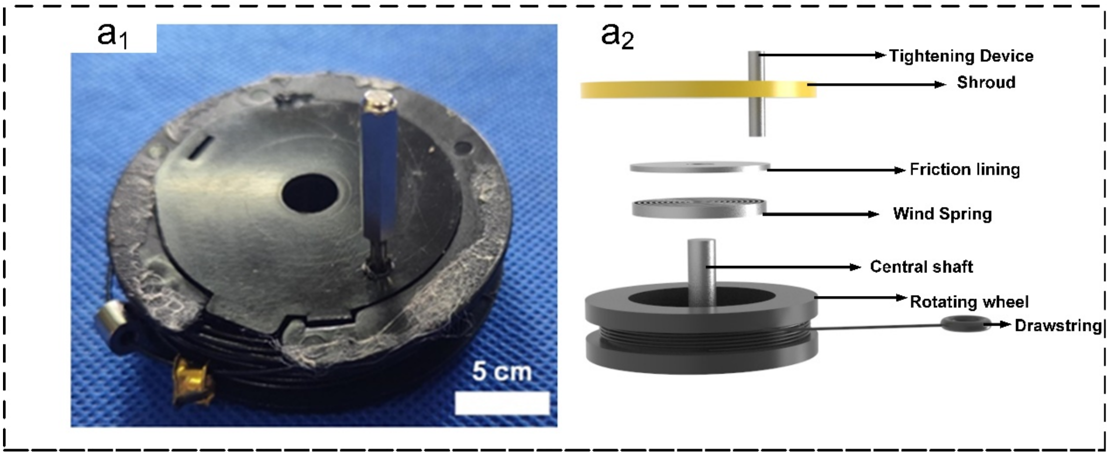

Figure S2. (a) MSTKJS-TENG physical image of the recoil starter, and (b) MSTKJS-TENG Internal structure diagram of the pulley.

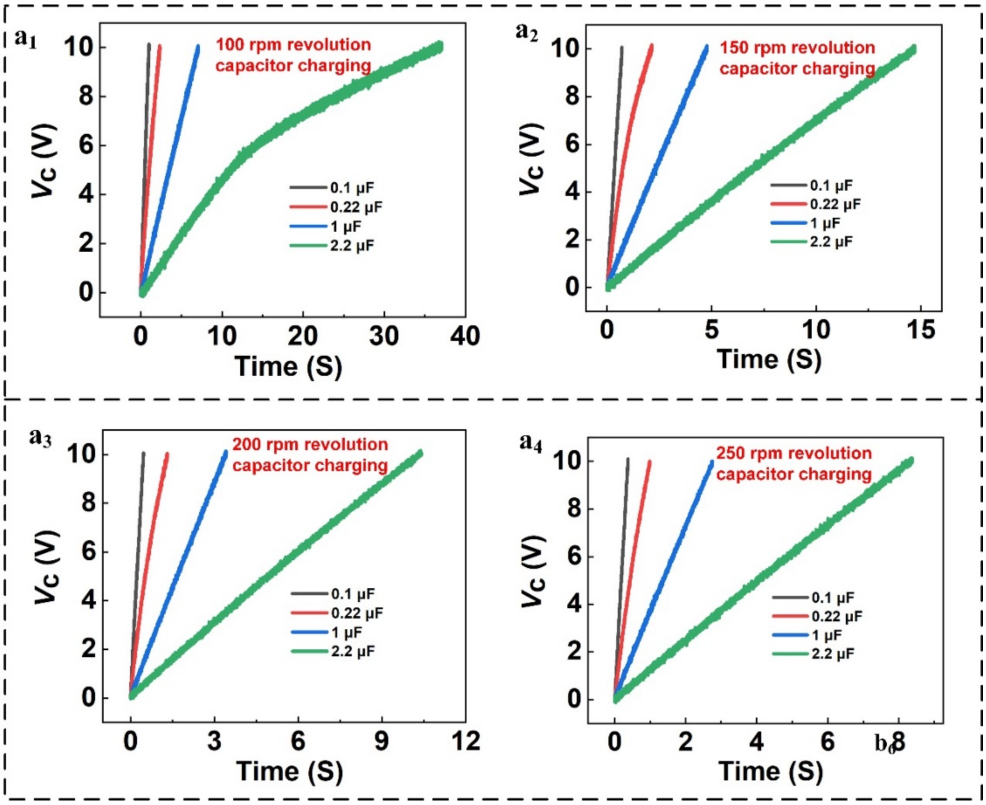

Figure S3. Voltage profiles demonstrating recharge patterns among diverse RPM/Capacitance configurations: (a_1) charging mode curve at 100 RPM, (a_2) charging mode curve at 150 RPM, (a_3) charging mode curve at 200 RPM, and (a_4) charging mode curve at 200 RPM.

MUC module algorithm program
```c
#include "stm32f1xx_hal.h"
#include <string.h>
#include <stdio.h>

// Global Variables
volatile uint32_t pulse_count = 0;            // Pulse Counter
volatile uint32_t group_count = 0;            // Group Counter
uint32_t n_value = 5;                         // Default: Every n pulses count as 1
uint8_t led_state = 0;                        // LED State

// Function Declarations
void SystemClock_Config(void);
static void MX_GPIO_Init(void);
static void MX_USART2_UART_Init(void);
void process_command(char *cmd);

UART_HandleTypeDef huart2;

int main(void)
{
    HAL_Init();
    SystemClock_Config();
    MX_GPIO_Init();
    MX_USART2_UART_Init();

    char welcome_msg[] = "\r\nSquare Wave Counter Started Successfully!\r\n";
    char prompt_msg[] = "Please enter command: set n=value or get\r\n";
    HAL_UART_Transmit(&huart2, (uint8_t*)welcome_msg, strlen(welcome_msg), 1000);
    HAL_UART_Transmit(&huart2, (uint8_t*)prompt_msg, strlen(prompt_msg), 1000);

    while (1)
    {
        // Process serial commands
        if (HAL_UART_Receive(&huart2, (uint8_t*)rx_buffer, 1, 10) == HAL_OK)
        {
            process_uart_data(rx_buffer[0]);
        }

        HAL_Delay(100);
    }
}
```

```
// System Clock Configuration
void SystemClock_Config(void)
{
	RCC_OscInitTypeDef RCC_OscInitStruct = {0};
	RCC_ClkInitTypeDef RCC_ClkInitStruct = {0};

	__HAL_RCC_PWR_CLK_ENABLE();

__HAL_PWR_VOLTAGESCALING_CONFIG(PWR_REGULATOR_VOLTAGE_SCALE1
);

	RCC_OscInitStruct.OscillatorType = RCC_OSCILLATORTYPE_HSI;
	RCC_OscInitStruct.HSIState = RCC_HSI_ON;
	RCC_OscInitStruct.HSICalibrationValue = RCC_HSICALIBRATION_DEFAULT;
	RCC_OscInitStruct.PLL.PLLState = RCC_PLL_ON;
	RCC_OscInitStruct.PLL.PLLSource = RCC_PLLSOURCE_HSI_DIV2;
	RCC_OscInitStruct.PLL.PLLMUL = RCC_PLL_MUL16;
	HAL_RCC_OscConfig(&RCC_OscInitStruct);

	RCC_ClkInitStruct.ClockType                                        =
RCC_CLOCKTYPE_HCLK|RCC_CLOCKTYPE_SYSCLK

|RCC_CLOCKTYPE_PCLK1|RCC_CLOCKTYPE_PCLK2;
	RCC_ClkInitStruct.SYSCLKSource = RCC_SYSCLKSOURCE_PLLCLK;
	RCC_ClkInitStruct.AHBCLKDivider = RCC_SYSCLK_DIV1;
	RCC_ClkInitStruct.APB1CLKDivider = RCC_HCLK_DIV2;
	RCC_ClkInitStruct.APB2CLKDivider = RCC_HCLK_DIV1;
	HAL_RCC_ClockConfig(&RCC_ClkInitStruct, FLASH_LATENCY_2);
}

// GPIO Initialization
static void MX_GPIO_Init(void)
{
	GPIO_InitTypeDef GPIO_InitStruct = {0};

	__HAL_RCC_GPIOA_CLK_ENABLE();
	__HAL_RCC_GPIOC_CLK_ENABLE();
	__HAL_RCC_GPIOB_CLK_ENABLE();

	// PA0 - External Interrupt Input
	GPIO_InitStruct.Pin = GPIO_PIN_0;
	GPIO_InitStruct.Mode = GPIO_MODE_IT_RISING;    // Rising Edge Trigger
	GPIO_InitStruct.Pull = GPIO_PULLDOWN;
	HAL_GPIO_Init(GPIOA, &GPIO_InitStruct);
```

```
    // PC13 - LED Output
    GPIO_InitStruct.Pin = GPIO_PIN_13;
    GPIO_InitStruct.Mode = GPIO_MODE_OUTPUT_PP;
    GPIO_InitStruct.Pull = GPIO_NOPULL;
    GPIO_InitStruct.Speed = GPIO_SPEED_FREQ_LOW;
    HAL_GPIO_Init(GPIOC, &GPIO_InitStruct);

    // Configure External Interrupt
    HAL_NVIC_SetPriority(EXTI0_IRQn, 0, 0);
    HAL_NVIC_EnableIRQ(EXTI0_IRQn);
}

// USART2 Initialization
static void MX_USART2_UART_Init(void)
{
    huart2.Instance = USART2;
    huart2.Init.BaudRate = 115200;
    huart2.Init.WordLength = UART_WORDLENGTH_8B;
    huart2.Init.StopBits = UART_STOPBITS_1;
    huart2.Init.Parity = UART_PARITY_NONE;
    huart2.Init.Mode = UART_MODE_TX_RX;
    huart2.Init.HwFlowCtl = UART_HWCONTROL_NONE;
    huart2.Init.OverSampling = UART_OVERSAMPLING_16;
    HAL_UART_Init(&huart2);
}

// External Interrupt Callback Function
void HAL_GPIO_EXTI_Callback(uint16_t GPIO_Pin)
{
    if (GPIO_Pin == GPIO_PIN_0)
    {
        pulse_count++;

        // Every n pulses count as 1
        if (pulse_count >= n_value)
        {
            group_count++;
            pulse_count = 0;

            // LED Blink Indication
            led_state = !led_state;
            HAL_GPIO_WritePin(GPIOC, GPIO_PIN_13, led_state ? GPIO_PIN_SET :
GPIO_PIN_RESET);
```

```
        }
    }
}

// Serial Receive Buffer
uint8_t rx_buffer[64];
uint8_t rx_index = 0;

// Serial Data Processing
void process_uart_data(uint8_t data)
{
    if (data == '\r' || data == '\n')
    {
        if (rx_index > 0)
        {
            rx_buffer[rx_index] = '\0';
            process_command((char*)rx_buffer);
            rx_index = 0;
        }
    }
    else if (rx_index < sizeof(rx_buffer) - 1)
    {
        rx_buffer[rx_index++] = data;
    }
}

// Command Processing Function
void process_command(char *cmd)
{
    char response[100];

    if (strncmp(cmd, "set n=", 6) == 0)
    {
        uint32_t new_n = atoi(cmd + 6);
        if (new_n > 0 && new_n <= 1000000)    // Limit range
        {
            n_value = new_n;
            pulse_count = 0;    // Reset counter
            sprintf(response, "Set successfully: n=%lu\r\n", n_value);
        }
        else
        {
            sprintf(response, "Error: n value must be between 1-1000000\r\n");
        }
```

```
    }
    else if (strcmp(cmd, "get") == 0)
    {
        sprintf(response, "Current Status:\r\n");
        HAL_UART_Transmit(&huart2, (uint8_t*)response, strlen(response), 1000);

        sprintf(response, "    Pulse Count: %lu\r\n", pulse_count);
        HAL_UART_Transmit(&huart2, (uint8_t*)response, strlen(response), 1000);

        sprintf(response, "    Group Count: %lu\r\n", group_count);
        HAL_UART_Transmit(&huart2, (uint8_t*)response, strlen(response), 1000);

        sprintf(response, "    n Value: %lu\r\n", n_value);
        HAL_UART_Transmit(&huart2, (uint8_t*)response, strlen(response), 1000);

        return;
    }
    else if (strcmp(cmd, "reset") == 0)
    {
        pulse_count = 0;
        group_count = 0;
        sprintf(response, "Counter reset\r\n");
    }
    else
    {
        sprintf(response, "Unknown command\r\nAvailable commands: set n=value, get, reset\r\n");
    }

    HAL_UART_Transmit(&huart2, (uint8_t*)response, strlen(response), 1000);
}

// External Interrupt Service Function
void EXTI0_IRQHandler(void)
{
    HAL_GPIO_EXTI_IRQHandler(GPIO_PIN_0);
}
```

Usage Instructions

1. Hardware Connection
- Square Wave Signal → PA0 Pin.
- LED Indicator → PC13 Pin.

- Serial Port → USART2 (PA2-TX, PA3-RX).

2. Serial Commands

Send the following commands via serial port:

- `set n=5` - Set every 5 pulses to count as 1.
- `get` - Get current counting status.
- `reset` - Reset the counter.

3. Features

- Real-time square wave pulse counting.
- Generates one group count for every n pulses.
- LED indicates group count.
- Supports runtime modification of n value.
- Monitoring and configuration via serial port.

4. Example

When setting `n=5`:

- Input 5 square waves → Group Count = 1.
- Input 15 square waves → Group Count = 3.
